# Wireless and Health

**Published:** 1924-11

**Authors:** 


### WIRELESS AND HEALTH.

### TALKS WITH THE MILLION.

Our object, said Sir George Newman the other day to his audience of some millions, is not the entertainment of those listening-in, but the improvement of the health of the nation. We have before made brief reference to the fact that the Chief Medical Officer of the Ministry of Health, with a true instinct for progress and for the value of up-to-date methods, had been broadcasting talks on public health. Sir George Newman has now, we understand, entered upon a series of talks for the purpose of establishing among his millions of hearers a good understanding and an enlightened opinion and of evoking a definite response.

### A Partnership in Health.

He invites a partnership in this matter between the public and the Ministry of Health. The first definite step in this direction is to point out, as Sir George Newman has done, the various aspects of public health and then to invite those who are listening to tell the Chief Medical Officer what are the subjects that they would wish him to talk to them about. The next step, of course, is for his hearers to act upon the advice given to them and to inspire others with the same ideas. We have little doubt that the public desire practical advice given in simple, non-technical language on those every-day matters of hygiene, sanitation and nutrition which, properly observed, make up an enormous sum of human happiness and, when neglected, entail so much suffering and depression.

### Personal Hygiene and Health Week.

In a further talk, in which Sir George Newman brought to notice the subject of Health Week, he indicated the very matters to which we have just referred as those most urgent in relation to personal health. First, habits of personal cleanliness, cleanliness of the home, including feeding utensils and milk; cleanliness of body, including skin, mouth, teeth and digestive system. Secondly, the breathing of fresh air—after all, the air we breathe supplies the blood with its oxygen, to be carried to all parts of the body; and the blood is the life. Vast reform is needed in the ventilation of bedrooms, living rooms, offices and workshops. Thirdly, good food. We do not take sufficient care to select suitable and nourishing food. It is not a question of money; we spend money on what is not bread. It is a question of selection, and this means knowledge. We would urge Sir George Newman and those whose assistance he enlists in his organised series of talks to see that definite practical instruction is given in regard to this very important matter of right food and its right preparation. We want to get away from the views of food faddists and avoid being caught in the meshes of food advertisers. A clear statement is badly needed in A B C language, and there is no one who is more competent to do this than Sir George Newman.

### Clear Thinking Wanted.

In order that there may be clear thinking upon the subjects which could promptly be handled in the talks broadcasted as part of the series which the Chief Medical Officer is arranging, he has indicated five definite aspects of the subject which is comprised in the term Public Health. First, there is a Science of public health, what Professor Starling has called the Wisdom of the Body—what it is, how it works, what it requires for health and so on. Closely akin to this are the nature of disease, the causes of disease, how the human body may be so kept and safeguarded as to resist disease and last a long time; what are the effects of environment and food, of sunlight and air, of rest and work; what are the natural laws, at the back of all these things, which are embraced by the science of public health. Secondly there is the Art of Preventive Medicine; the public ought to know about sanitation, water supply, food control, maternity and child welfare, rheumatism and cancer, tuberculosis, factory hygiene, the school medical service, industrial welfare. Thirdly, there is the fascinating aspect of the subject which may be called the Conquests of Preventive Medicine. There was a time, says Sir George Newman, when leprosy was prevalent in England. Where is it now? It is almost an extinct disease in this country. Plague used to sweep through our English towns and decimate their populations. It used to hold up all commerce and industry and put an end even to our politics! But it has gone. Why has it gone? Smallpox did the same. Hardly a family was exempt; the face with pocks was a common sight in the street. Now it is a very rare disease and usually much milder than in the old days. Why is that?

### A Wembley Reflection.

We may learn, too, how the white man has won the control of the tropics, not by mere invasion, but by the conquest of disease—malaria, yellow fever, etc. When, says Sir George Newman, I stand in the Health Section at the Wembley Exhibition, I ask myself this question: "Is not this little section the secret of the expansion of the British Empire? Is it not the physique of the British people and their control of disease which has made the Empire possible?" Fourthly, there is a Social and Economic Aspect of public health—its relation to population and its distribution to the social habits and customs of the people, to their family life and laws, to alcoholism, to poverty, to occupation, trade and industry, to housing and the lay-out of cities, to labour and unemployment.

### Builders of Empire.

Then, finally, there is a history of those great founders of modern medicine, whose names ought to be household words—Harvey, Hunter, Jenner, Simpson, Pasteur, Lister, Simon and Manson. These men were master-builders in the commonwealth of learning and human service. We have little doubt that Sir George Newman will find an effective response if he continues to present to the public interesting word pictures showing every aspect of preventive medicine. The individual listener-in can gradually be brought to realise that he has an individual responsibility in this matter.

